# Principles of bacteriostatic and bactericidal antibiotics at subinhibitory concentrations

**DOI:** 10.1128/mbio.02066-25

**Published:** 2025-10-17

**Authors:** Elizabeth Vaisbourd, David Shaanan Glass, Yifan Yang, Avi Mayo, Anat Bren, Uri Alon

**Affiliations:** 1 Department of Molecular Cell Biology, Weizmann Institute of Science 34976, Rehovot, Israel; 2 Center for Interdisciplinary Studies, Westlake University 557712, Hangzhou, Zhejiang, China; 3 Sagol Institute for Longevity Research, Weizmann Institute of Science 34976, Rehovot, Israel; Universite Libre de Bruxelles, Gosselies, Belgium

**Keywords:** antibiotics, bacterial physiology, bactericidal, bacteriostatic

## Abstract

Subinhibitory antibiotic exposures are common in clinical and environmental contexts, yet their effects on bacterial growth dynamics remain incompletely understood. We studied the temporal response of *Escherichia coli* to a panel of bactericidal (“cidal”) and bacteriostatic (“static”) antibiotics at sub-minimum inhibitory concentrations (sub-MIC). We uncover a sharp dynamical distinction between the two classes. Bacteriostatic antibiotics reduce the initial growth rate in a dose-dependent manner, similar to a nutrient starvation response. In contrast, bactericidal antibiotics do not alter initial growth rates—cells continue to grow as fast as untreated cells—until an abrupt slowdown in growth rate. The onset of the slowdown occurs earlier with increasing concentration, suggesting a damage accumulation mechanism leading to a lethal threshold. Cidals also show a steeper concentration-response curve. We propose that bacteria respond to cidal antibiotics with a “grow-fast-then-crash” strategy that is adaptive for transient lethal threats, whereas static antibiotics trigger stress adaptation and slower growth. While clinical outcomes of statics and cidals are similar at full inhibitory concentrations, these sub-MIC dynamical signatures could influence treatment outcomes. Our findings offer a dynamic framework for antibiotic classification and raise new questions about how bacteria respond to sublethal antibiotic stress.

**IMPORTANCE:**

Understanding how antibiotics influence bacterial growth dynamics at subinhibitory concentrations is crucial for interpreting treatment outcomes. Traditional classifications into bacteriostatic and bactericidal agents rely on endpoint measurements that obscure temporal behaviors critical to bacterial physiology and survival. By analyzing high-resolution growth trajectories of *Escherichia coli* exposed to 15 antibiotics, our study reveals that bacteriostatics and bactericidals differ fundamentally not only in their outcomes but in their dynamic modes of action. These insights challenge static minimal inhibitory concentration/minimal bactericidal concentration definitions and offer a new, time-resolved framework for antibiotic classification—one that captures the kinetics of damage and response. Our findings have implications for clinical treatment strategies and for understanding how bacteria survive in sublethal antibiotic environments.

## INTRODUCTION

Antibiotics have improved health and lifespan over the past century (1). They can be classified in many ways, including by their origin (natural or synthetic), spectrum of activity (narrow or broad), mechanism of action, clinical role, and resistance risk (2, 3). Mechanistically, antibiotics target diverse bacterial functions such as translation, transcription, cell wall synthesis, DNA maintenance, and metabolism (2, 4, 5).

One particularly common classification relies on treatment outcomes—bactericidal antibiotics (“cidals”), which kill bacteria, and bacteriostatic antibiotics (“statics”), which inhibit their growth (4). In some cases, static and cidal antibiotics target the same biological process, such as translation. Although it was once thought that cidal antibiotics are clinically superior, this notion has been challenged; treatment success depends on numerous factors beyond the cidal/static distinction (6, 7).

Despite its widespread use, the binary distinction between bacteriostatic and bactericidal antibiotics remains ambiguous, especially at subinhibitory concentrations. The formal definition hinges on the ratio between the minimal bactericidal concentration (MBC) and the minimal inhibitory concentration (MIC), which are traditionally determined by colony-forming unit counting and turbidity, respectively. MIC is defined as the lowest concentration preventing visible growth after an overnight culture, while MBC is the lowest concentration that reduces the viable population by at least 99.9%. An antibiotic is considered cidal if MBC/MIC < 4, and static otherwise (8).

In practice, the classification of an antibiotic can vary depending on the bacterial species, the experimental context, or the specific strain tested (9). The classic methods used to define MIC and MBC may fail to distinguish between slow death and slow growth, particularly at subinhibitory concentrations, where net population change may not reflect the underlying dynamics. Moreover, the cidal/static classification does not reveal why some antibiotics lead to bacterial death while others inhibit growth. Thus, current definitions based on MIC and MBC ratios do not capture dynamic bacterial responses, potentially limiting clinical effectiveness and our understanding of bacterial survival strategies.

A clear phenotypic distinction between cidal and static antibiotics, especially at subinhibitory concentrations, could help reveal the physiological strategies used by bacteria to survive (10). Measuring growth patterns over time across a range of antibiotic concentrations can provide temporal and quantitative response features that remain invisible when classifying antibiotics based on two concentrations and a single endpoint.

In this study, we explored whether bactericidal and bacteriostatic antibiotics differ in their dynamic impacts on bacterial growth at subinhibitory concentrations. By growing *Escherichia coli* (*E. coli*) at many subinhibitory concentrations across a panel of 15 antibiotics, we find that cidal and static antibiotics induce qualitatively different growth trajectories. Static antibiotics immediately reduce the growth rate in a dose-dependent manner and allow bacteria to proliferate until reaching the carrying capacity. In contrast, cidal antibiotics do not affect the initial growth rate but cause an abrupt reduction of growth after a dose-dependent time. We suggest that the accumulation of antibiotic-dependent damage causes the reduction of growth rate and propose a mathematical model to explain the time dynamics and concentration dependence. Furthermore, cidal antibiotics generally have a steeper concentration-response curve and higher halfway points in terms of their MIC than static antibiotics. We discuss the utility of these growth responses in maximizing fitness in short versus long-term stress conditions.

## RESULTS

### Subinhibitory antibiotics show class-specific differences in *E. coli* growth dynamics

We sought to characterize *E. coli* growth dynamics in a wide panel of antibiotics with distinct mechanisms of action and static/cidal classification (Table 1). To this end, we measured the growth curves of exponentially growing *E. coli* MG1655 at high temporal resolution (7–10 minutes) in a robotic system immediately following treatment with a range of subinhibitory antibiotic concentrations (11–14). MIC was defined as the lowest concentration at which the growth curve plateaued after a brief growth phase or failed to reach an optical density (OD) of 0.05 after 10 h of treatment (see Table S1 for MIC values). We validated the static/cidal outcomes by standard definitions, measuring the MBC/MIC ratio using plate assays (8) (Table S1, see Materials and Methods).

**TABLE 1:** Mechanistic and phenotypic characteristics of antibiotics used in this study

Antibiotic	Chemical nature	Target and downstream effects	Antibacterial activity in gram-negative bacteria
Ampicillin	β-Lactam (5)	Penicillin-binding protein. Cell wall synthesis inhibitor (5, 15).	Bactericidal (15)
Chloramphenicol	Phenicol (5)	Ribosome large subunit. Inhibits translation by preventing incoming aa-tRNA binding (16, 17).	Bacteriostatic (17)
Ciprofloxacin	Fluoroquinolone (5)	DNA gyrase. Causes dsDNA breaks (18).	Bactericidal (18)
Doxycycline	Tetracycline (5)	Ribosome small subunit (5). Prevents tRNA binding to the ribosome and inhibits protein synthesis (19).	Bacteriostatic (19)
Erythromycin	Macrolide (5)	Binds the large ribosomal subunit, blocks nascent peptide exit channel (20, 21).	Bacteriostatic (22)
Fosfomycin	Phosphonic antibiotic (23)	Inhibits synthesis of peptidoglycan precursors, leading to loss of cell wall integrity and lysis (5, 23, 24).	Bactericidal (5)
Gentamicin	Aminoglycoside (5)	16S rRNA of the ribosome small subunit. Causes mistranslated proteins and membrane potential destabilization (25).	Bactericidal (5)
Nalidixic acid	Fluoroquinolone (18)	DNA gyrase. Causes dsDNA breaks (18).	Bactericidal (18)
Nitrofurantoin	Nitrofuran (26)	Nitrofurantoin is reduced by flavoproteins to highly reactive electrophilic molecules that indiscriminately attack essential macromolecules (DNA, ribosomes, and cell wall) (26).	Bactericidal at high concentrations but bacteriostatic at low concentrations (26, 27)
Rifampicin	Rifamycin (5)	rpoB (β-subunit) of the RNA polymerase. Inhibits transcription by steric blocking of elongating RNA (28).	Bactericidal (29, 30). Not affecting *E. coli* (31). Static, with higher MIC for gram-negative than gram-positive (32) Static (33).
Spectinomycin	Aminoglycoside (5)	Ribosome small subunit. Inhibits the translocation of mRNA and tRNA. Does not cause mistranslation (5, 34).	Bacteriostatic (5)
Streptomycin	Aminoglycoside (5)	Ribosome small subunit (35). Causes mistranslation by tRNA mismatching and protein aggregates (5, 36, 37).	Bactericidal (37)
Tetracycline	Tetracycline (5)	Ribosome small subunit. Tetracycline inhibits translation by preventing the binding of aminoacyl-tRNA to the A site (38).	Bacteriostatic (17, 19)
Thiolutin	Dithiolopyrrolones (39)	Inhibitor of mRNA synthesis (chain elongation) (40).	Bacteriostatic (30, 39)
Trimethoprim	Trimethoprim (41, 42)	Dihydrofolate reductase (folA). Inhibits folate synthesis (5).	Bacteriostatic (41)

**Fig 1:**
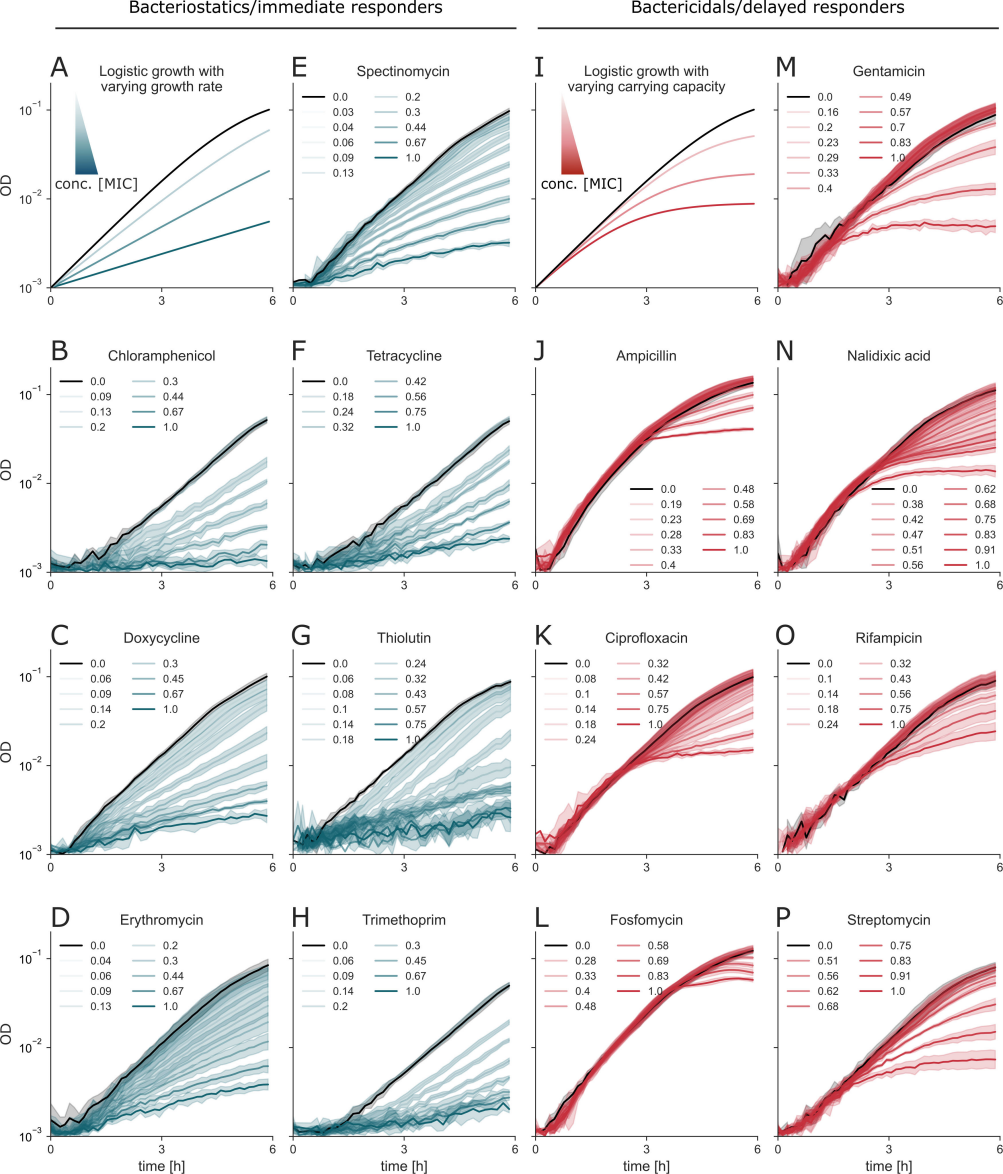
The growth traces of *E. coli* under bacteriostatic antibiotics are distinct from those under bactericidal treatment. (**A and I**) Schematics showing the effects of bacteriostatic and bactericidal antibiotics on *E. coli* growth rate and yield, respectively. (**B–H**) Growth curves under bacteriostatic treatment. (**J–P**) Growth curves under bactericidal treatment. Treatment concentrations are normalized relative to the MIC and expressed as multiples of the MIC (Table S1). Data are presented as the mean ± SD of five to six technical replicates. See Fig. S2 for two more biological replicates.

The OD traces show two distinct behaviors. The seven static antibiotics show log-linear curves with slopes that deviate from the untreated curve at very early time points in a dose-dependent manner (schematic representation in Fig. 1A; data in Fig. 1B through H). At long times, biomass reaches the carrying capacity (Fig. S1). The seven cidal antibiotics show more complex growth dynamics. The growth curves are very similar to the untreated curve for the first 1.5–3 h, followed by a reduced growth rate that decreases in a dose-dependent manner (schematic representation in Fig. 1I; data in Fig. 1J through P). Similar growth trajectories that discriminate between cidal and static antibiotics were also observed with the BW25113 strain of *E. coli* (Fig. S3).

**Fig 2:**
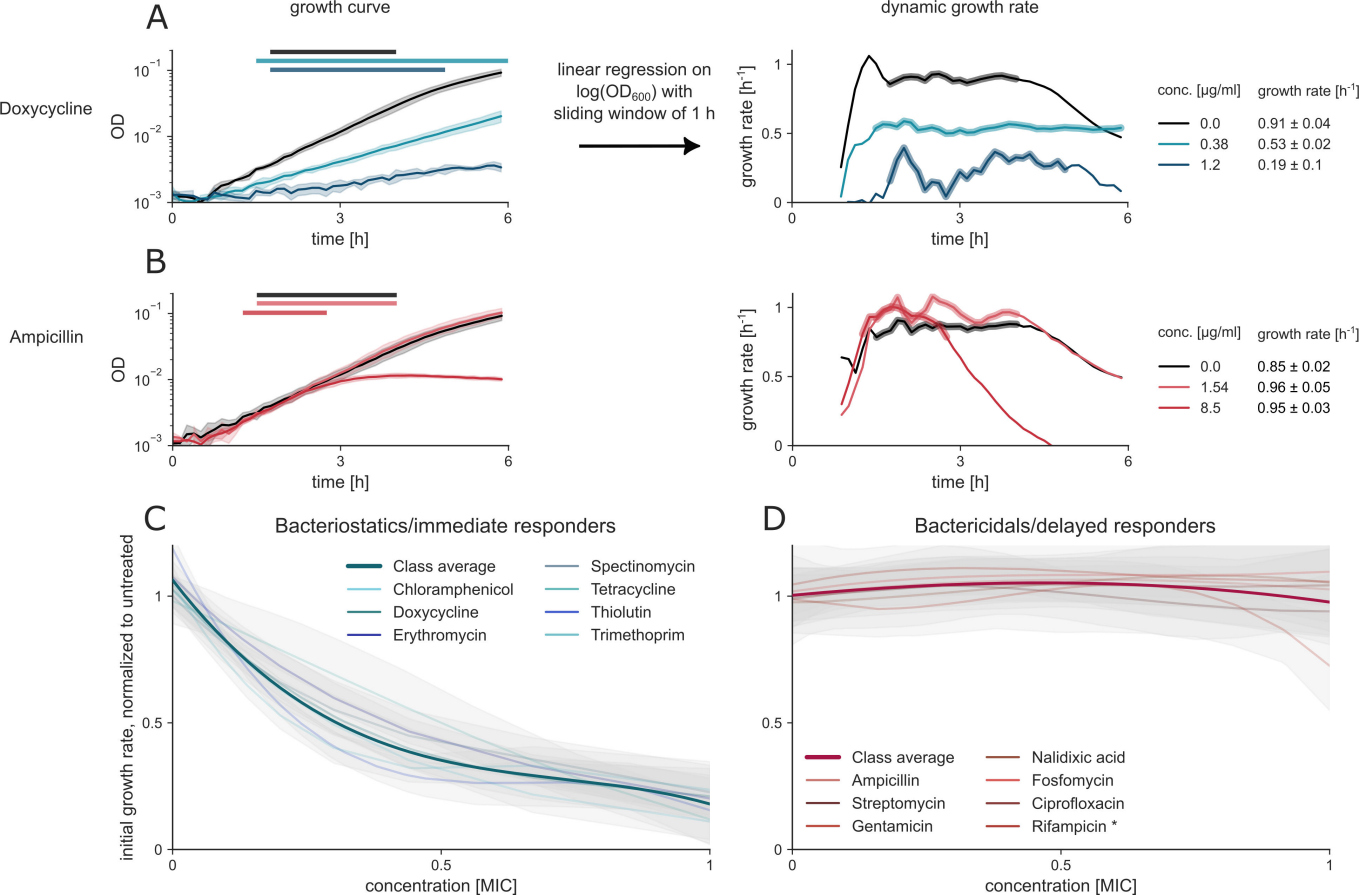
Static antibiotics reduce the initial growth rate in a concentration-proportional manner, whereas cidal antibiotics do not affect the initial growth rate (**A and B**). Representative growth curves for ampicillin (**A**) and doxycycline (**B**), with horizontal bars indicating the time intervals used for growth rate estimation. In both cases, growth rates were calculated by fitting a linear regression to log-transformed OD values and averaged across the selected time interval for each treatment. (**C**) Bacteriostatic antibiotics exhibit a dose-dependent inhibitory effect on growth. (**D**) Bactericidal antibiotics, however, exert their effect largely independently of concentration, maintaining a similar initial growth rate to untreated controls. Rifampicin is an outlier, being a delayed responder in terms of growth rate, but bacteriostatic in the MBC/MIC assay. Antibiotic concentrations are normalized to the MIC, and growth rates are normalized to untreated cultures. Concentration-response curves by drug represent spline fits across three biological replicates (each with four to six technical replicates). Class-level curves are the average of bootstrapped drug curves (see Fig. S4 for raw data).

We quantified the initial growth rate (Fig. 2A and B), highlighting these two distinct phenotypes: a growth rate that drops with concentration (Fig. 2C) and a growth rate that remains similar to untreated, even at concentrations close to the MIC (Fig. 2D).

These two phenotypes correspond to the bacteriostatic and bactericidal classification (Table 1), with one possible exception (rifampicin). All seven static antibiotics showed a dose-dependent drop in initial growth rate (immediate responders), and seven cidal antibiotics showed no effect on initial growth rate (Fig. 2C and D). Rifampicin was a partial exception: while its growth response was delayed in a cidal-like manner, plating assays suggested a more static-like behavior (MBC/MIC > 4).

Thus, we conclude that most antibiotics can be sorted into cidal or static based on growth rate response at sub-MIC concentrations.

### Cidal antibiotics have steeper concentration dependence and a higher halfway point than static antibiotics

**Fig 3:**
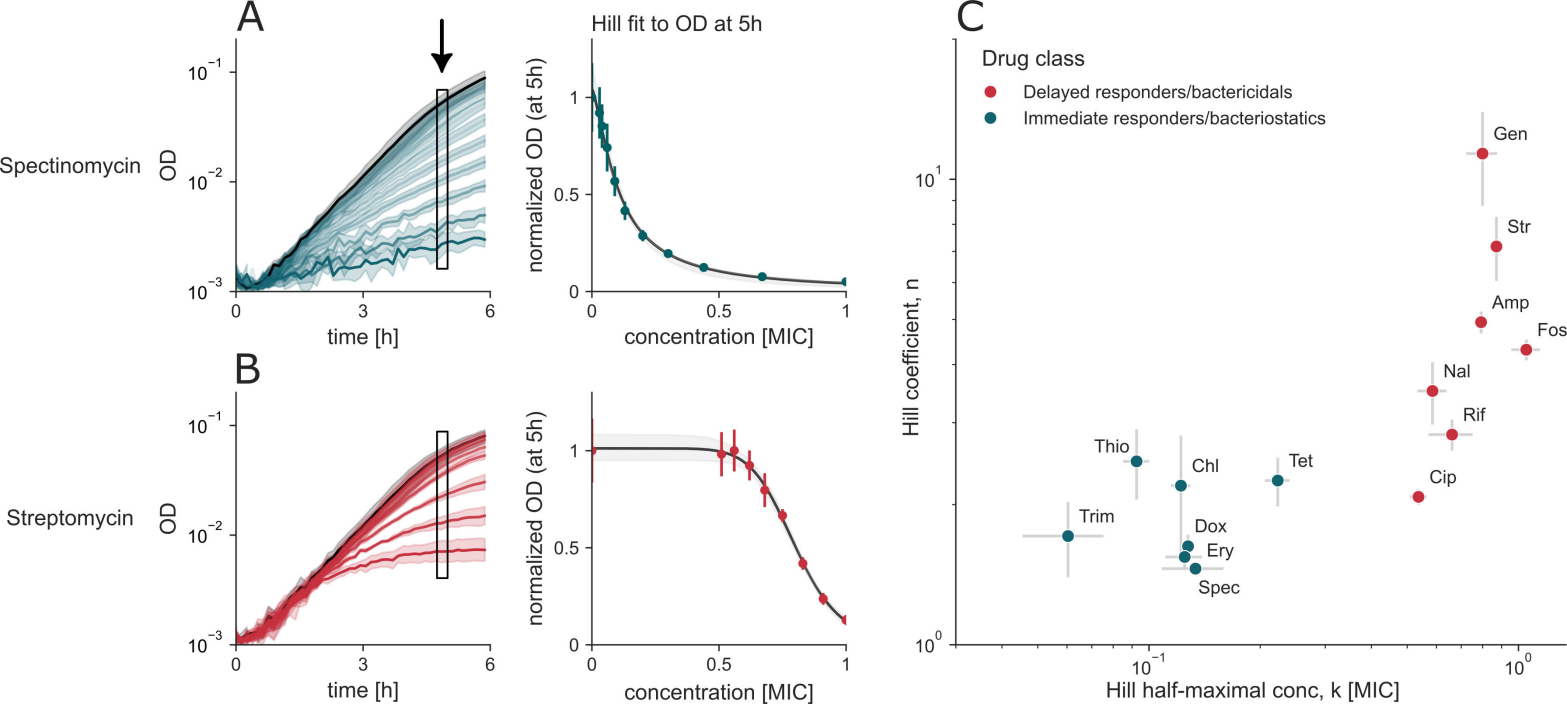
Cidal antibiotics are more cooperative and have higher halfway points than static antibiotics. (**A and B**) Growth traces of spectinomycin (**A**) and streptomycin (**B**). Error bars denote the SD of technical replicates, and the gray shaded region around the Hill fit indicates 95% CI. (**C**) Hill coefficients for each drug class are separable with a wide margin and cluster together. *k* is expressed in units of MIC (see Materials and Methods and Table S2), error bars show SEM of three biological repeats, each with four to six technical replicates. Trim, trimethoprim; Dox, doxycycline; Ery, erythromycin; Spec, spectinomycin; Thio, thiolutin; Chl, chloramphenicol; Tet, tetracycline; Cip, ciprofloxacin; Rif, rifampicin; Nal, nalidixic acid; Amp, ampicillin; Fos, fosfomycin; Str, streptomycin; and Gen, gentamicin.

We next examined the concentration dependence of antibiotic effects by measuring the bacterial yield after 5 h of treatment across a range of concentrations (Fig. 3A and B). All seven static antibiotics exhibited a gradual dose-dependent decrease in yield, consistent with a Michaelis-Menten-like behavior, characterized by Hill coefficients in the range of ~1–2. In contrast, all seven cidal antibiotics displayed a steeper, cooperative concentration-response relationship, with Hill coefficients ranging from ~2 to 9 (Fig. 3C; Fig. S5).

Cidal antibiotics also exhibited higher half-maximal effective concentrations (*k*), expressed in units of MIC, compared to static antibiotics (Fig. 3C). These trends were robust to the sampling time: analyzing the data at 4–6 h yielded similar Hill coefficients and halfway points (Fig. S6).

### Growth from different initial dilutions shows duration-dependent effects in cidal antibiotics and OD-dependent effects in static antibiotics

**Fig 4:**
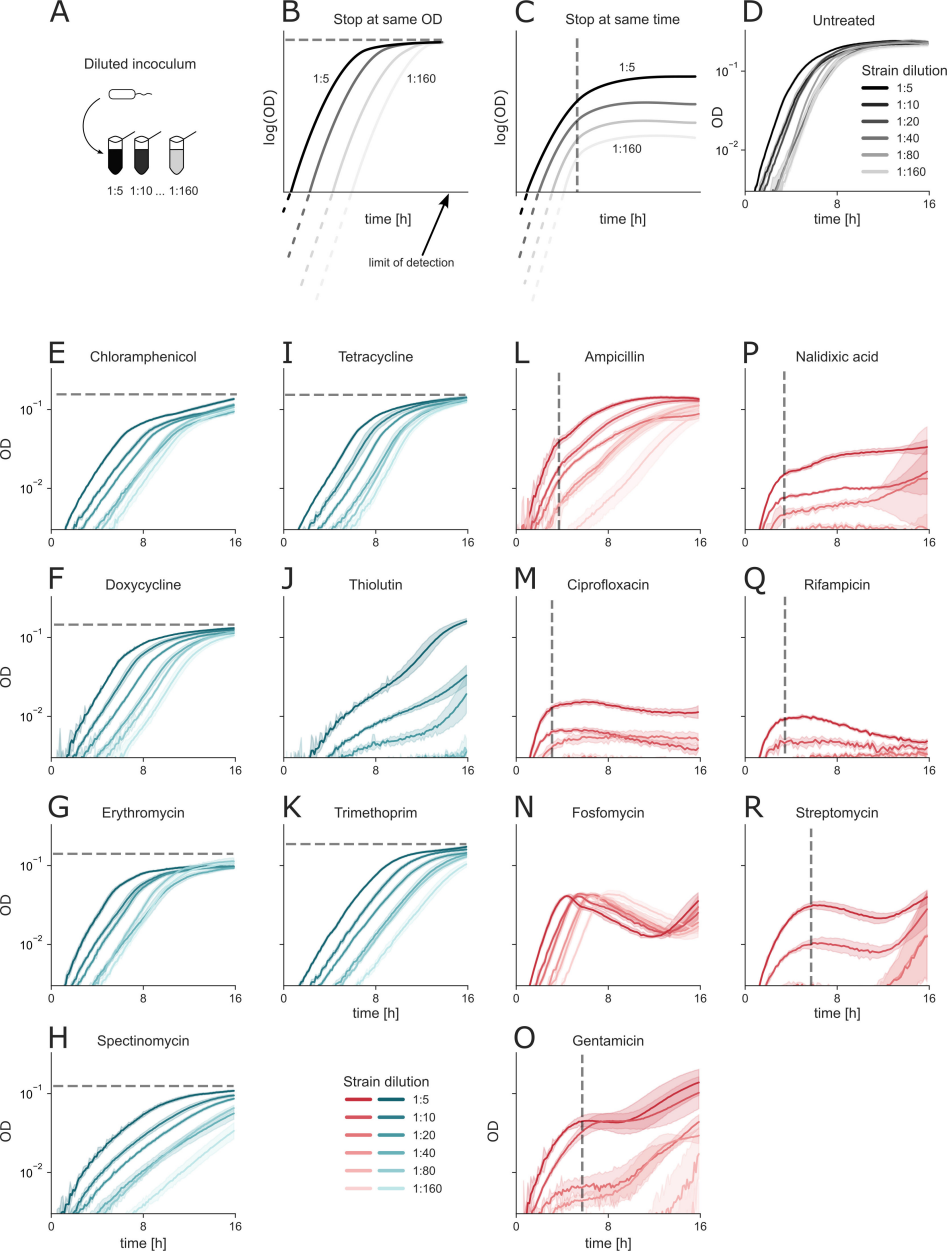
Different initial inocula show duration-dependent effects in cidal antibiotics and OD-dependent effects in static antibiotics. (**A**) *E. coli* cultures were diluted to six inoculum concentrations (1:5, 1:10, 1:20, 1:40, 1:80, and 1:160 from exponential pre-growth). (**B**) Schematic representation of the expected result if growth stops at a given OD. (**C**) Schematic representation of growth stops due to treatment duration. (**D**) Untreated cultures reach the same OD. (**E–K**) Static antibiotics stop at a given OD. (**L–R**) Cidal antibiotics stop after a given treatment duration. Gray dashed lines indicate the OD or time dependency on the growth dynamics. Data are presented as the mean ± SD of four to six technical replicates.

The onset of the abrupt growth arrest in cidal antibiotics could be driven by either bacterial density or by the duration of exposure to treatment. To distinguish these two possibilities, we compared growth using an initial inoculum size spanning a 32-fold range in bacterial density (Fig. 4A). We hypothesized that if growth is density-dependent, all cultures would reach the same optical density (Fig. 4B). In contrast, if the antibacterial effect depends on treatment duration, growth curves would bend at the same time regardless of initial density (Fig. 4C).

We observe that an untreated culture and cultures treated with most static antibiotics follow the same growth pattern as the density-dependent model, regardless of the initial inoculum (Fig. 4D through K). In contrast, cultures treated with cidal antibiotics growing from different inocula stop growing after approximately the same treatment duration, reaching different yields (Fig. 4L through R).

One exception to the static group is thiolutin, with growth trajectories that cannot be classified into either of the groups (Fig. 4J). Another exception is the cidal antibiotic fosfomycin, showing an OD-dependent behavior with an increased crash time for a smaller inoculum size, unlike other antibiotics (Fig. 4N). Following the cultures over a prolonged duration of treatment with cidal antibiotics (more than ~10 h) reveals that some exhibit a secondary regrowth behavior (Fig. 4N, O, P, and R; Fig. S7).

We conclude that the abrupt stop of bacterial growth in cidal antibiotics depends on treatment duration. We hypothesize that the time delay in the growth stop reflects the duration it takes for antibiotic-inflicted damage to become substantial enough to cause a growth halt. On the other hand, growth under static treatment is limited by the growth conditions’ maximal yield (carrying capacity).

### Nitrofurantoin shows a growth signature that is neither cidal nor static

To understand cases where the cidal/static dichotomy has difficulty in classifying antibiotics, we also studied an antibiotic that has a mixed static/cidal categorization in the literature—nitrofurantoin. It is reported to have static effects at low concentrations and cidal effects at high concentrations (26, 27). Nitrofurantoin has a pleiotropic mechanism of action, affecting multiple metabolic and macromolecular processes in the cell (26) (Table 1).

**Fig 5:**
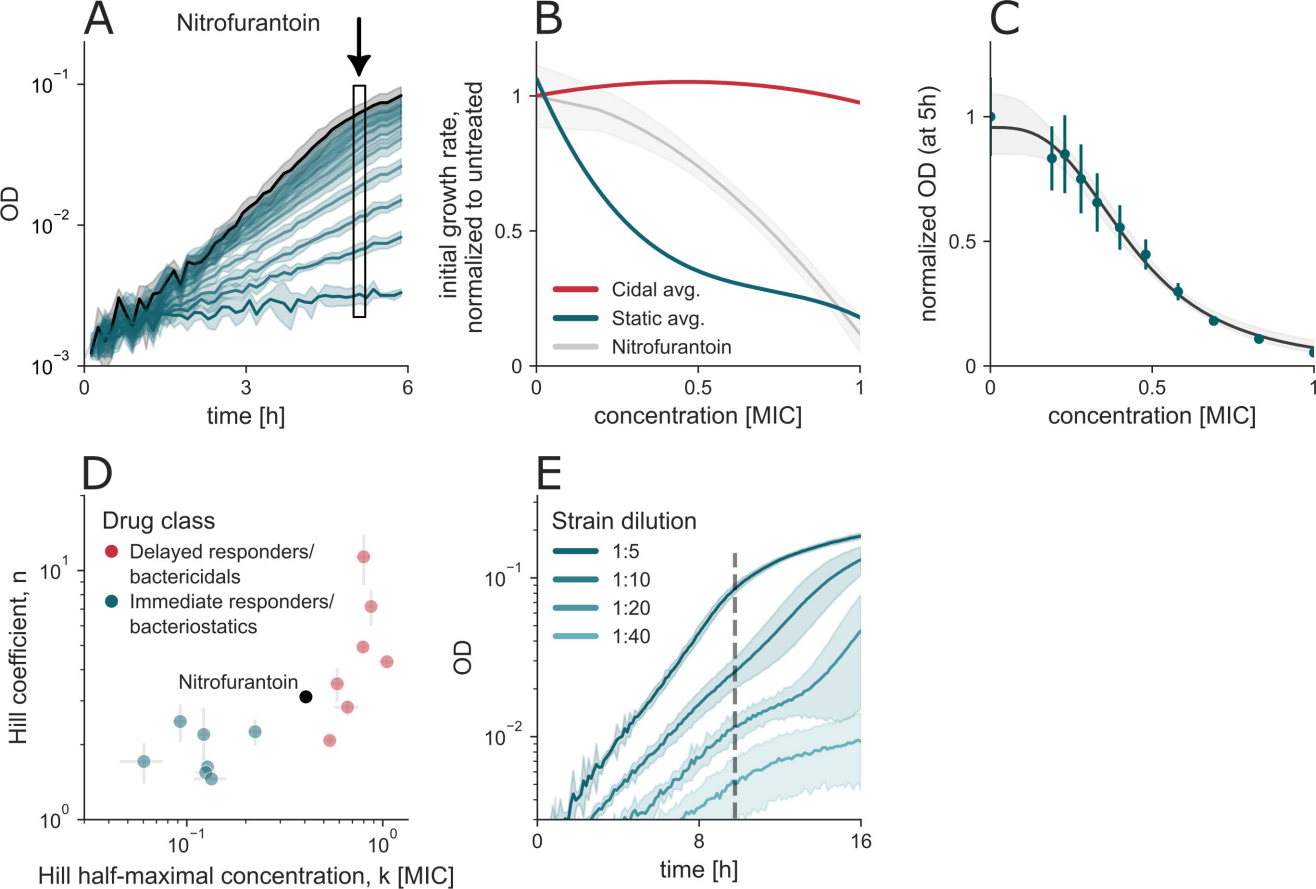
Nitrofurantoin has a mixed cidal and static signature. (**A**) Growth curves under nitrofurantoin treatment in sub-MIC concentrations. (**B**) Nitrofurantoin’s growth rate decreases with treatment concentration. (**C**) OD_600_ after 5 h of treatment as a function of treatment concentration. Error bars denote the SD of technical replicates, and the gray shaded region around the Hill fit indicates 95% CI. (**D**) Hill coefficients for nitrofurantoin fall between the cidal and static groups. (**E**) *E. coli* growth under nitrofurantoin treatment exhibits both a slow growth rate and a bend, characteristics of both static and cidal antibiotics, respectively.

We find that nitrofurantoin shows a growth signature that is neither cidal nor static. Growth curves in sub-MIC concentrations appear qualitatively bacteriostatic (Fig. 5A). Quantification of the initial growth rate and density at 5 h, however, does not match either category. The Hill coefficient resides between those of the cidal and static classes (Fig. 5B through D). When cultured from varying initial inocula, we observe a mixed phenotype (Fig. 5E). When subjected to the MBC/MIC test, nitrofurantoin is lethal to cells at 4×MIC after 24 h of treatment. These observations confirm that a drug classified as mixed cidal/static cannot be classified into either group in our growth-based analysis.

### Mathematical model of damage accumulation predicts the onset of slowdown in growth rate in cidal antibiotics

To establish a connection between antibiotic concentration and growth dynamics, we developed a mathematical model for the abrupt slowdown in growth rate phenotype of cidal antibiotics that incorporates a latent variable, damage, as a mediating factor. Since the time to slowdown onset depends on the duration of treatment, we posit that cidal antibiotics generate intracellular damage, *y*, which stops rapid growth when it crosses a threshold $y_{c}$ (43, 44).

We assume that the damage production rate depends on the growth rate *r*, and that damage is diluted by biomass growth. We also assume that the damage production rate is linear in antibiotic concentration *c*. Thus, damage production rises as a function of antibiotic concentration and is diluted by the growth rate *r*.


(1) $$\dot{y}=r(y)\;(\alpha c-y).$$

The bacterial biomass *N* grows with a logistic behavior, with the growth rate *r* and carrying capacity *K*.


(2) $$\dot{N}=r(y)\;N\left(1-\frac{N}{K}\right).$$

Finally, we use our observation of a constant growth rate at early times to specify the dependence of growth rate on damage as constant $r_{0}$ when damage is below a threshold $y_{c}$, and a Hill-like decrease with cooperativity *n* when damage exceeds the threshold


(3) $$r(y)=\left\{ \begin{array}{ll} r_{0}, & \;\text{if }y<y_{c}\\ \frac{2r_{0}}{1+{\left(\frac{y}{y_{c}}\right)}^{n}}, & \;\text{if }y\geq y_{c} \end{array}\right..$$

**Fig 6:**
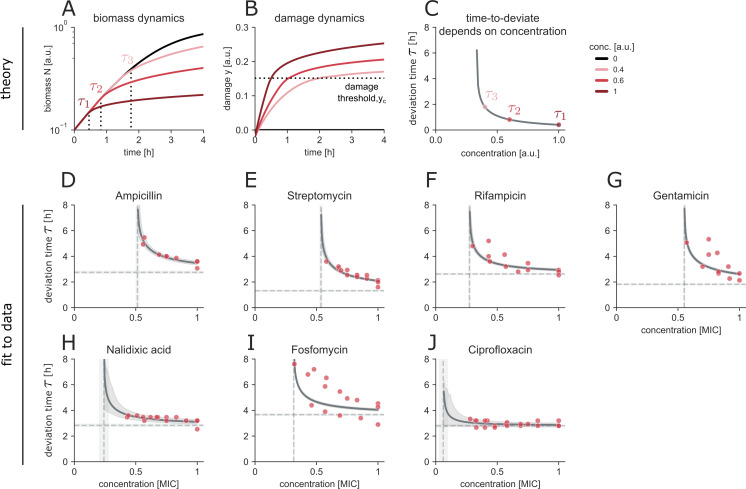
Time to deviate from the untreated curve depends on the treatment concentration of cidal antibiotics and is explained by linear accumulation of damage. (**A**) Simulated biomass accumulation dynamics of untreated (black) and treated (shades of red) bacterial cultures. Time points of deviation from the untreated curve are annotated τ. (**B**) Simulation of damage accumulation dynamics in bacterial culture. (**C**) Times of deviation as a function of the treatment concentration, with annotated $\tau_{1-3}$ corresponding to 6A. (**D–J**) Fits of the model (gray) to the data (red) (*N* = 3 biological replicates). The shaded area indicates 90% CI on the fitted parameters. Horizontal lines indicate the fitted $\tau_{0}$, the minimal duration for a deviation to occur, and the vertical lines indicate the fitted $\frac{y_{c}}{\alpha }$, the minimal antibiotic concentration to elicit a deviation from the untreated curve.

Qualitatively, the model (equations 1-3) captures the growth curves under cidal treatment (Fig. 6A), namely the initial exponential phase and a subsequent slowdown in growth rate. The model also predicts the concentration-dependent damage accumulation for (Fig. 6B).

We can determine the onset time τ when $y(\tau )=y_{c}$ by analytically solving with the initial condition $y(\tau_{0})=0$, where $\tau_{0}$ is the time needed for the antibiotic to start producing damage:


(4) $$\tau =\tau_{0}+\frac{1}{r_{0}}\ln\left(\frac{c}{\;c-\frac{y_{c}}{\alpha }}\right).$$

Simulating the timepoints of deviation resulted in concentration-dependent behavior (Fig. 6A and C). We further determined the model parameters using the observed initial growth rate $r_{0}$ and by fitting the parameters $\tau_{0}$ and $\frac{y_{c}}{\alpha }$ for each antibiotic. The model captured the onset of slowdown in growth rate well, with an RMS error of 0.24 h (Fig. 6D through J; Table S3 for parameters and fit metrics), outperforming a baseline model (*P* = 0.02; see Materials and Methods).

One interesting prediction is that the onset time diverges at a finite concentration ($c^{*}=\frac{y_{c}}{\alpha }$), around half of the MIC for most antibiotics. Thus, the model captures the absence of effect on the growth curve at low concentrations. This is because dilution by growth outpaces the damage production and keeps damage below the threshold. Nalidixic acid and ciprofloxacin—both DNA-gyrase inhibitors—displayed stopping times that were largely concentration independent (Fig. 6H and J), suggesting a distinct physiological response that merits further investigation.

### A trade-off between speed and repair may shape the response

**Fig 7:**
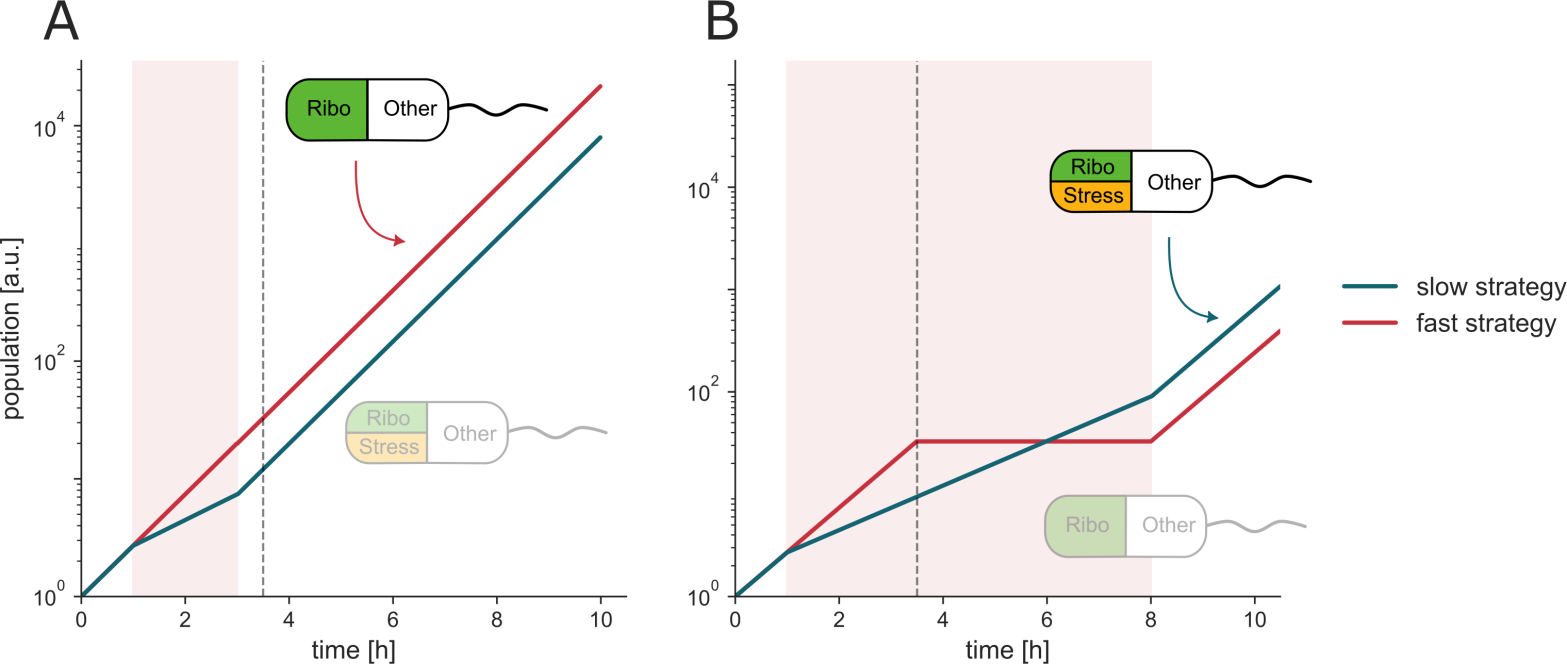
Grow-as-fast-as-possible outperforms a grow-slow-and-repair strategy when lethal stressors are brief. (**A**) A short antibiotic pulse (red band, 1–4 h) ends before the deviation time τ. The ribosome-rich fast-growing population (red), therefore, accumulates more biomass than the slow, repair-biased population (teal). (**B**) When the duration of stress is longer than the deviation time, damage collapses the fast population, and the slow strategy prevails. Insets schematically show how each cell partitions its resources among growth (ribosome “Ribo,” green), stress mitigation (“Stress,” orange), and other, constant functions (white); the faded cartoon marks the losing strategy in each panel. See Materials and Methods for simulation information.

The sharp distinction between static and cidal growth responses raises a question: why do bacteria under cidal treatment continue growing rapidly and stop abruptly, rather than slowing their growth from the outset? We explore the potential evolutionary implications of this behavior through an example. Suppose that bacteria can take one of two growth strategies: grow-as-fast-as-possible or grow-slow-and-repair. In the fast strategy, cells devote a large fraction of their proteome to ribosomes, allowing rapid growth as in drug-free conditions but neglecting stress mitigation, which is detrimental in the long term. In contrast, the slow strategy allocates resources to repair or defense at the cost of a slower growth rate (11). We explored these strategies under cidal and static antibiotic stresses of different durations using simulations. Brief stresses, shorter than the time to deviate τ, provide an advantage to the grow-as-fast-as-possible strategy (Fig. 7A). Longer stress periods provide an advantage to the grow-slow-and-repair strategy, if at the end of the stress period, the slow strategy population outnumbers the fast strategy population (Fig. 7B). One may reason that the grow-as-fast-as-possible strategy has an advantage in a situation of lethal stressors of brief duration (less than $\tau_{0}$, which is about 2–3 h in the present data). Currently, however, we cannot distinguish between this evolved strategy and the possibility of a passive accumulation of damage, which leads to growth halt and death.

## DISCUSSION

We studied the growth dynamics of *E. coli* in response to a panel of 15 antibiotics at subinhibitory concentrations. We find a stark dynamical distinction of different growth features between bactericidal and bacteriostatic antibiotics. Cidal antibiotics do not affect the initial growth rate and then cause an abrupt reduction of growth, whereas static antibiotics decrease the initial growth rate in a dose-dependent manner. This finding is consistent with previous studies on specific antibiotics (30, 45–47). We found one antibiotic, nitrofurantoin, with a growth pattern that does not match either group, consistent with previous studies in which static and cidal effects have been shown. We further show that the time to crash in cidal antibiotics is time-dependent rather than OD-dependent. It would be interesting in future work to test whether this distinction extends to other gram-negative and/or gram-positive bacteria.

Taken together, these results support the notion that a treatment with cidal antibiotics results in damage accumulation that leads to death when the damage crosses a critical threshold. We propose a mathematical model that describes the temporal dynamics of antibiotic-inflicted damage in a dose-dependent manner and is diluted by cell division. This mechanism of death by cidal antibiotics is consistent with reactive oxygen species accumulation following treatment with a variety of cidal antibiotics (48, 49), misfolded protein accumulation following streptomycin treatment (50), and in starving *E. coli* (44). This mechanism might be affected by the nutritional conditions and metabolic rates, which are likely increased in order to compensate for the resources invested in repair, as shown for ampicillin in Mathiew et al*.* (51). In contrast to cidal antibiotics, static antibiotics may resemble starvation-like regulation of growth, which is titrated gradually with the restriction of cellular processes by subinhibitory antibiotic levels (30, 52).

We propose an evolutionary explanation, supported by a mathematical model, for the unchanged initial growth under cidal antibiotics. In this framework, a grow-as-fast-as-possible strategy is advantageous when the lethal stress is only transient. On the other hand, a slow-and-repair strategy can be more optimal if typical challenges in the environment are prolonged (longer than a few hours). These strategies might resemble the difference between low levels of rich nutrients, in which bacteria grow fast and then crash (12), to high levels of poor nutrients, in which the growth rate is reduced (53).

Clinical outcomes are often similar for bacteriostatic and bactericidal antibiotics (7, 8). Consistent with our finding, at the single-colony level, sub-MIC static treatment slows down the growth rate of all colonies, whereas cidal treatment allows escape of some colonies at sub-MIC levels (30). This may imply that in the sub-MIC regime, static antibiotics are more efficient than cidal ones. These differences at sub-MIC concentrations may matter clinically in cases when bacteria experience sub-MIC exposures in fluctuating environments and protected niches.

Future work can test mechanisms of the cidal “crash” and static growth reduction. Previous studies on antibiotic growth dynamics were carried out in a range of conditions, and testing the cidal-static distinction in a corresponding range of conditions is important for generalizability. Future studies can also explore the dynamics at the level of individual bacteria to supplement the present population data (44, 54). It would be important to test how these dynamics shape therapeutic success and immune responses. The present dynamical criterion may sharpen antibiotic classification and inspire further exploration into how bacteria survive low-concentration antibiotic challenges, with the hope of improving clinical outcomes.

## MATERIALS AND METHODS

### Strain

*E. coli* (MG1655, CGSC #6300) were grown overnight in M9 minimal medium (42 mM Na_2_HPO_4_, 22 mM KH_2_PO_4_, 8.5 mM NaCl, 18.5 mM NH_4_Cl, 2 mM MgSO_4_, and 0.1 mM CaCl) with 0.05% casamino acids and 0.2% (wt/vol) glucose (M9C + glucose) at 37°C and 250 rpm. For re-entry into the exponential growth phase, overnight cultures were diluted 1:300 into the same media and grown for 3–4 additional hours before treatment and initiation of measurements. Several experiments were done with BW25113 grown in M9 + 0.2% (wt/vol) glucose. We studied bacteria in defined media rather than MH to provide better control over medium composition. Standard MIC measurements are in MH (25, 55), and we measured MIC in defined media to be consistent with our measurements.

### Antibiotics

Fifteen antibiotics were used: nalidixic acid, nitrofurantoin, trimethoprim, streptomycin, spectinomycin, chloramphenicol, thiolutin, gentamicin, ampicillin, tetracycline, doxycycline, rifampicin, fosfomycin, erythromycin, and ciprofloxacin (Sigma-Aldrich). All antibiotics were dissolved in water and sterile-filtered except for trimethoprim and thiolutin (DMSO) and rifampicin (methanol). Stock solutions of antibiotics were stored at −20°C.

### Growth curve records

#### Growth under treatment with varying treatment concentrations

Antibiotics were diluted to 10 times the desired experimental concentrations manually or using a robotic liquid handler (FreedomEvo, Tecan), and 20 µL was distributed into 96-well plates by the robot. For each drug and concentration, five to six technical replicates were performed. Exponential phase cultures were diluted 1:10, and 180 µL was added to the plates. Then, 50 µL of mineral oil was added on top to avoid evaporation and transferred into an automated incubator, where they were grown at 37°C and shaken (6 Hz) for 15–50 h. Previous studies have demonstrated that the oil in our 96-well plate protocol does not interfere measurably with growth/gene expression (11–14). To measure growth, the plates were moved by a robotic arm once every 7–10 minutes into a multi-well multimode plate reader (Infinite M200Pro, Tecan), and the optical density, OD at 600 nm, was recorded. OD levels of ~0.05 were visible by eye.

#### Growth from varying inocula

The same protocol was followed in the experiments recording growth curves from different inoculum numbers, except that after pre-growth, cultures were diluted in ratios of 1:5, 1:10, 1:20, 1:40, 1:80, and 1:160. The cultures were treated with ampicillin (0.5 MIC), chloramphenicol (0.35 MIC), ciprofloxacin (0.9 MIC), doxycycline (0.4 MIC), erythromycin (0.35 MIC), fosfomycin (1.1 MIC), gentamicin (0.8 MIC), nalidixic acid (0.4 MIC), nitrofurantoin (1 MIC), rifampicin (1.3 MIC), spectinomycin (0.35 MIC), streptomycin (0.7 MIC), tetracycline (0.45 MIC), thiolutin (0.2 MIC), or trimethoprim (0.2 MIC). Culture conditions and OD recording were the same as for the growth curves.

### MIC determination

The MIC for “immediate responders” was defined as the lowest treatment concentration at which OD_600_ remained below 0.005 after 10 h. In contrast, the MIC for “delayed responders” was determined to be the lowest concentration that resulted in either a plateaued or declining growth curve at the second growth stage.

### Antibiotic classification

To distinguish between bactericidal and bacteriostatic antibiotics, exponential-phase *E. coli* cultures were treated with each drug at 4×MIC for 24 h. Before treatment, 100 µL of the untreated culture was plated, and at the end of the treatment, 100 µL of culture was serially diluted (10×) and plated on LB agar. The plates were incubated overnight at 37°C, and colony counts were compared before and after treatment. Antibiotics that reduced bacterial density by more than 3 log₁₀-fold were classified as bactericidal.

### Growth rate calculation

OD_600_ values were blanked by subtracting the average of the first four data points from the time series of the recorded values. Then, OD = 0.001 was added to all blanked values to account for the presence of bacteria below the detection limit. Dynamic growth rates were calculated using a rolling linear ordinary least squares regression on the time series, with a window of 60 minutes. The growth rate for each drug and concentration was calculated by averaging the dynamic growth rate across a manually selected time window in which the dynamic growth rate was approximately constant. The error for each growth rate was estimated with error propagation. To account for day-to-day variation, treated bacterial growth rates were normalized to the untreated bacterial growth rate in each experiment.

#### Growth rate dependence on concentration

We estimated dose-response curves for each drug by applying spline interpolation to normalized initial growth rate measurements across concentrations normalized to MIC. To account for experimental variability, we performed bootstrap resampling (*N* = 1,000) at the level of biological replicates. In each iteration, replicates were sampled with replacement, and values were perturbed using normally distributed noise with a standard deviation equal to the replicate-specific error. The perturbed values were averaged across replicates per concentration, and a UnivariateSpline (*s* = 1) was fit to the resulting curve.

For each drug, the mean interpolated curve was computed from the 1,000 bootstrapped splines, and the standard deviation across bootstraps was used to define a shaded uncertainty band (±1 SD). At the drug class level, we aggregated all bootstrapped drug curves to compute the class-average spline, with shading again representing ±1 SD across drugs. This approach captures biological variation and measurement uncertainty in the estimated growth response.

### Hill fit to OD at 5 h

We modeled the concentration-response relationship with a Hill function of the form


$$f\left(c\right)=\frac{\alpha }{1+{\left(\frac{c}{k}\right)}^{n}},$$

where *c* is the drug concentration, *α* is the maximum response, *k* is the half-maximal concentration, and *n* reflects the steepness. Data were aggregated by concentration and fitted using nonlinear least-squares regression. To estimate uncertainty, a nonparametric bootstrap was done, in which groups of concentration points were resampled with replacement while always including the conc = 0 MIC point; the 2.5th and 97.5th percentiles of the predicted responses were computed at each concentration to yield 95% confidence intervals.

### Modeling of biomass accumulation and damage accumulation

We defined time to deviate as the time point at which the growth rate has clearly transitioned away from the untreated growth rate. These time points were manually determined by visual inspection of the plotted growth curves, with three biological repeats per antibiotic. Equation 4, time-to-slowdown model


$$\tau =\tau_{0}+\frac{1}{r_{0}}\ln\left(\frac{c}{c-\frac{y_{c}}{\alpha }}\right)$$

was fitted for the parameters $\tau_{0}$, and $\frac{y_{c}}{\alpha }$ is the critical concentration (in units of MIC) below which the logarithmic term diverges, and $r=r_{0}=1$, the unchanged initial growth rate, normalized to the untreated growth rate. Parameters were estimated by nonlinear least squares with bounds $\tau_{0},\;\frac{y_{c}}{\alpha }>0$. For the simulations in Fig. 6A and B (equations 1-3), the following parameters were used: $r_{0}=1,k=1,\alpha =0.45,y_{c}=0.15,n=6,x_{0}=0.1.$

### Time-to-deviate model evaluation procedure

To quantify how well the mechanism-based time-to-deviate model predicts the duration of fast growth, we compared it with a naive mean model that simply returns the average duration observed in the training data. Evaluation was carried out independently for each antibiotic as follows.

#### Cross-validation design

We performed leave-one-replicate-out cross-validation for each of the seven drugs: two replicates were used for training and the held-out replicate for testing, yielding three test splits per drug. We computed the mean-squared error (units = h²) for every test set between the predicted and observed duration. Using a two-sided Wilcoxon signed-rank test, we tested the null hypothesis that the median difference is zero. Our analysis showed significantly lower mean-squared error (Wilcoxon signed-rank test, *n* = 21, two-sided *P* = 0.022; significance was declared at *α* = 0.05).

### Simulation of fast and slow growth strategies

To explore how different bacterial resource allocation strategies perform under stress, we developed a simple model of population growth dynamics during a single cycle of stress followed by recovery. Two fixed strategies were considered. In the fast strategy, cells prioritize rapid growth during stress and recovery but lack stress management. Under stress, these cells initially grow but deviate after a fixed delay τ. In the slow strategy, cells invest in stress response systems at the cost of slower growth. This strategy maintains reduced but sustained growth during stress and resumes fast growth post-stress. Simulations were implemented by integrating bacterial populations over time using


(5) $$N\left(t+\Delta t\right)=N\left(t\right)\;e^{r\left(t\right)\;\Delta t},$$

where the growth rate *r*(*t*) depends on the current phase (stress or recovery). For both strategies, growth in stress-free conditions continues at *r* = 1. During stress, fast strategy cells grow at *r* = 1, whereas slow strategy cells grow at *r* = 0.5. Each simulation began with *N* = 1 and progressed through a drug-free phase, one stress period (2 or 7 h) with τ=2.5, and a recovery period. Population trajectories were calculated numerically using Euler integration with a time step Δt=0.1. The population size was used as a proxy for fitness.

### Software

All calculations were performed in Python 3.10 using Numpy 2.2.4, pandas 2.2.3, and statsmodels 0.14.2. Figures were prepared in Python and edited in Inkscape.

## SUPPLEMENTAL MATERIAL

Supplemental figures and tables mbio.02066-25-s0001.pdf Figures S1-S7; Tables S1-S3.

ASM does not own the copyrights to Supplemental Material that may be linked to, or accessed through, an article. The authors have granted ASM a non-exclusive, world-wide license to publish the Supplemental Material files. Please contact the corresponding author directly for reuse.
