# Supplementary material for: Principles of bacteriostatic and bactericidal antibiotics at subinhibitory concentrations
Source: mBio. 2025 Oct 17;16(11):e02066-25. doi: 10.1128/mbio.02066-25 (PMC12607625; doi:10.1128/mbio.02066-25)
Supplement: Supplemental figures and tables — Figures S1-S7; Tables S1-S3. [file mbio.02066-25-s0001.pdf]

Principles of bacteriostatic and bactericidal antibiotics at subinhibitory concentrations

Supplementary Information

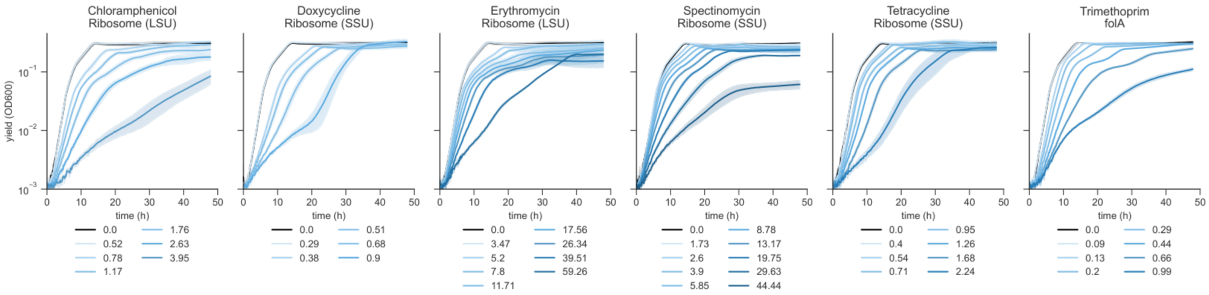

Figure S1. Long-term growth of *E. coli* treated with bacteriostatic antibiotics in M9C+glucose.

Exponential-phase *E. coli* were treated with sub-inhibitory doses of bacteriostatic antibiotics, and their growth curves were recorded once in 10 minutes for 48 h. Concentrations are indicated in µg/ml, except for ciprofloxacin, where it is ng/µl.

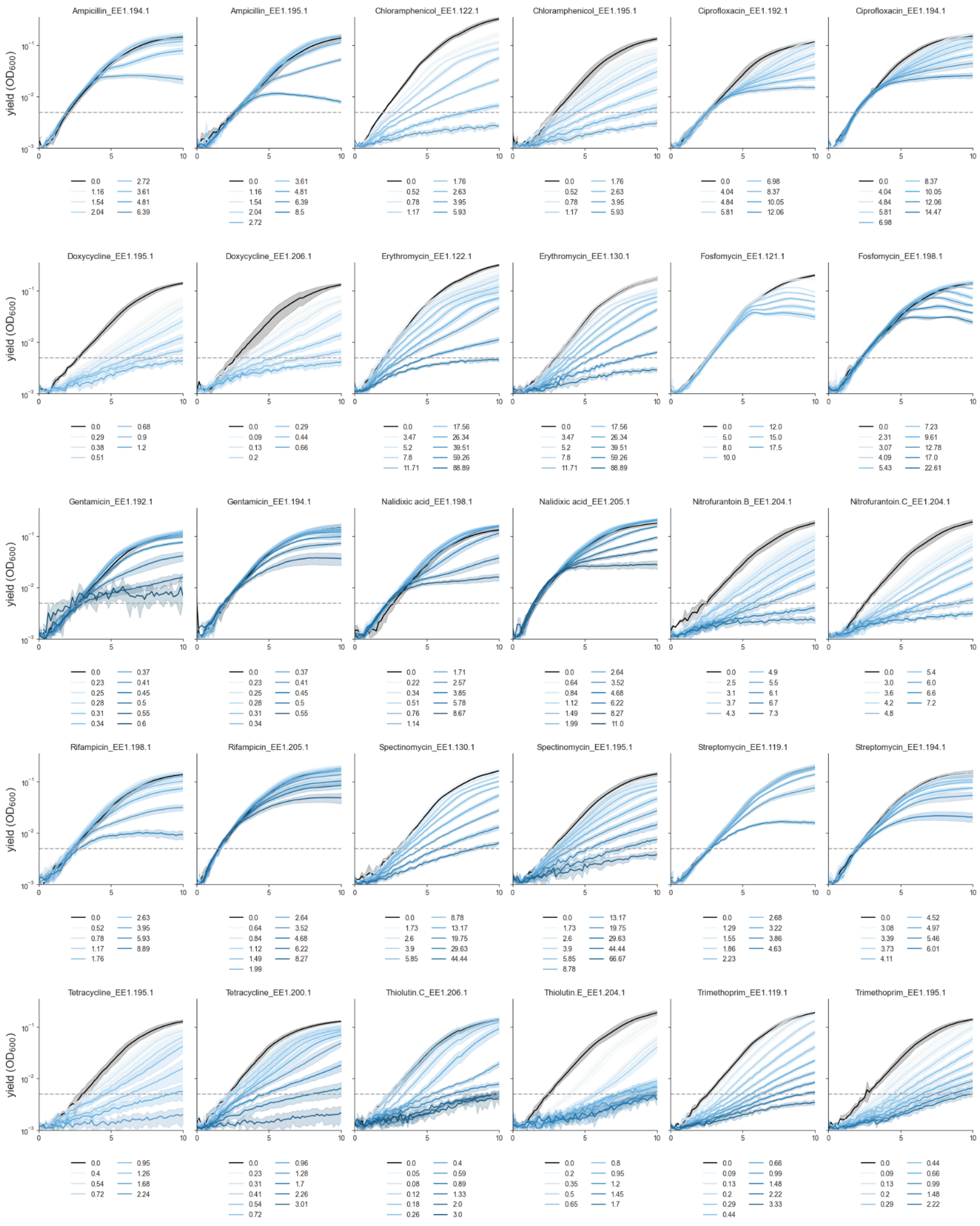

Figure S2. Two other biological repeats of the growth curves of the MG1655 strain in M9C+glucose.

Concentrations are indicated in µg/ml, except for ciprofloxacin, where it is ng/µl. 4-6 technical replicates per curve. Shown are treatments with concentrations smaller than or equal to the MIC in that experiment.

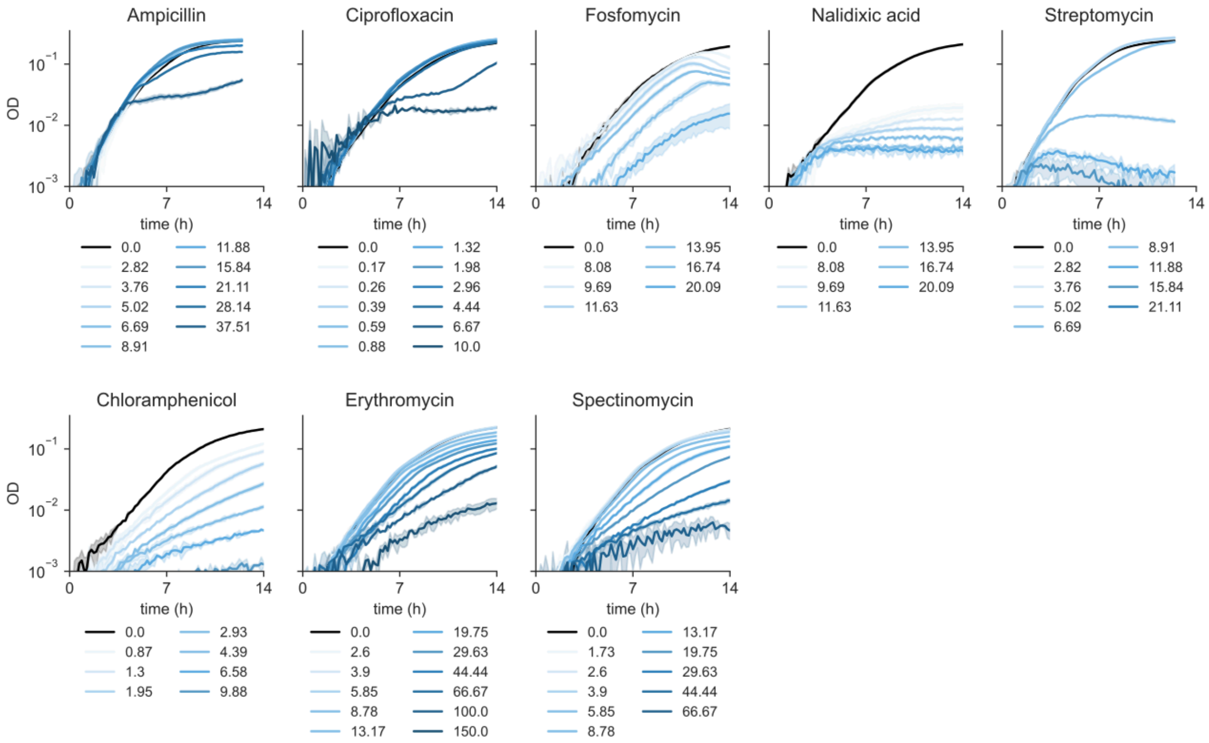

Figure S3. Growth trajectories of the BW25113 strain in a panel of 5 bactericidal and 3 bacteriostatic antibiotics, in M9+glucose.

Data are presented as means for 4-6 technical replicates per curve. Concentrations are indicated in µg/ml, except for ciprofloxacin, where it is ng/µl.

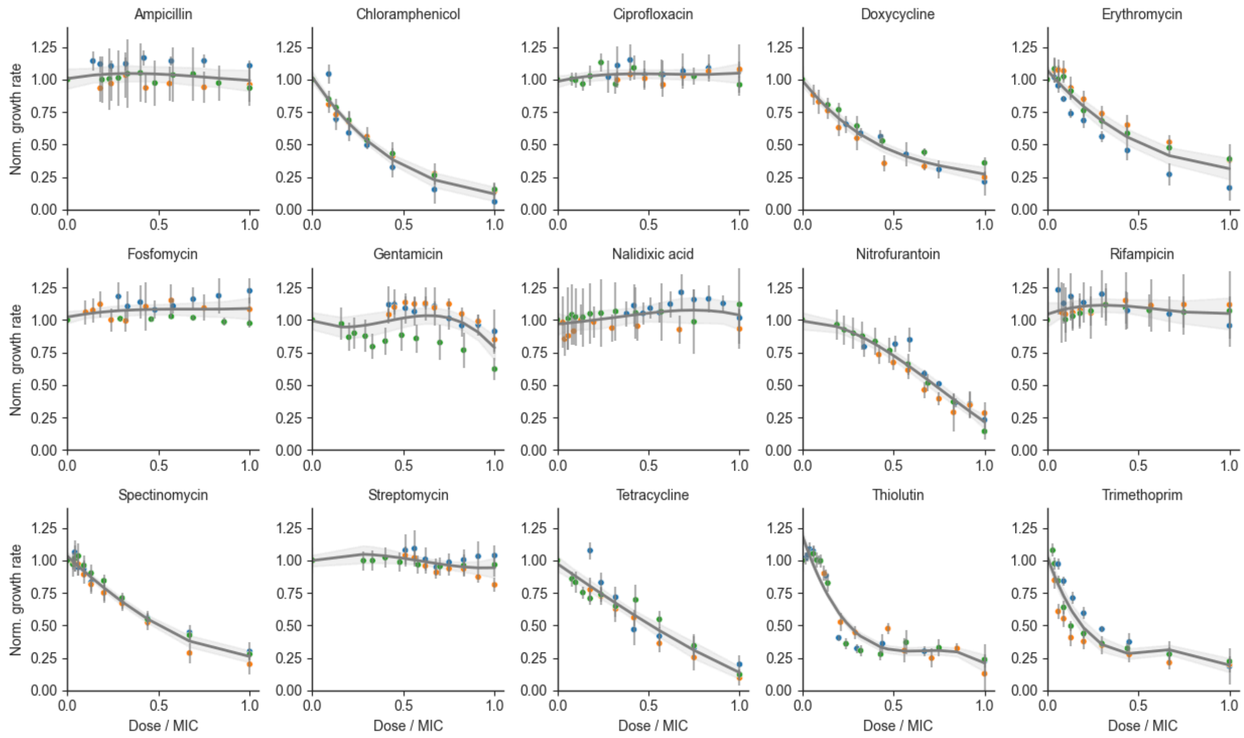

Figure S4. Growth rates of biological replicates.

Growth rates are normalized to the growth rate of the untreated control, and the treatment concentration is normalized to MIC.

Each color denotes a biological replicate, and error bars denote SD across 4-6 technical replicates.

For each drug, the curve represents a spline interpolation of bootstrapped samples (N=1000), where biological replicates were resampled with replacement and measurement noise was incorporated using replicate-specific error estimates. The shaded region represents ± SD.

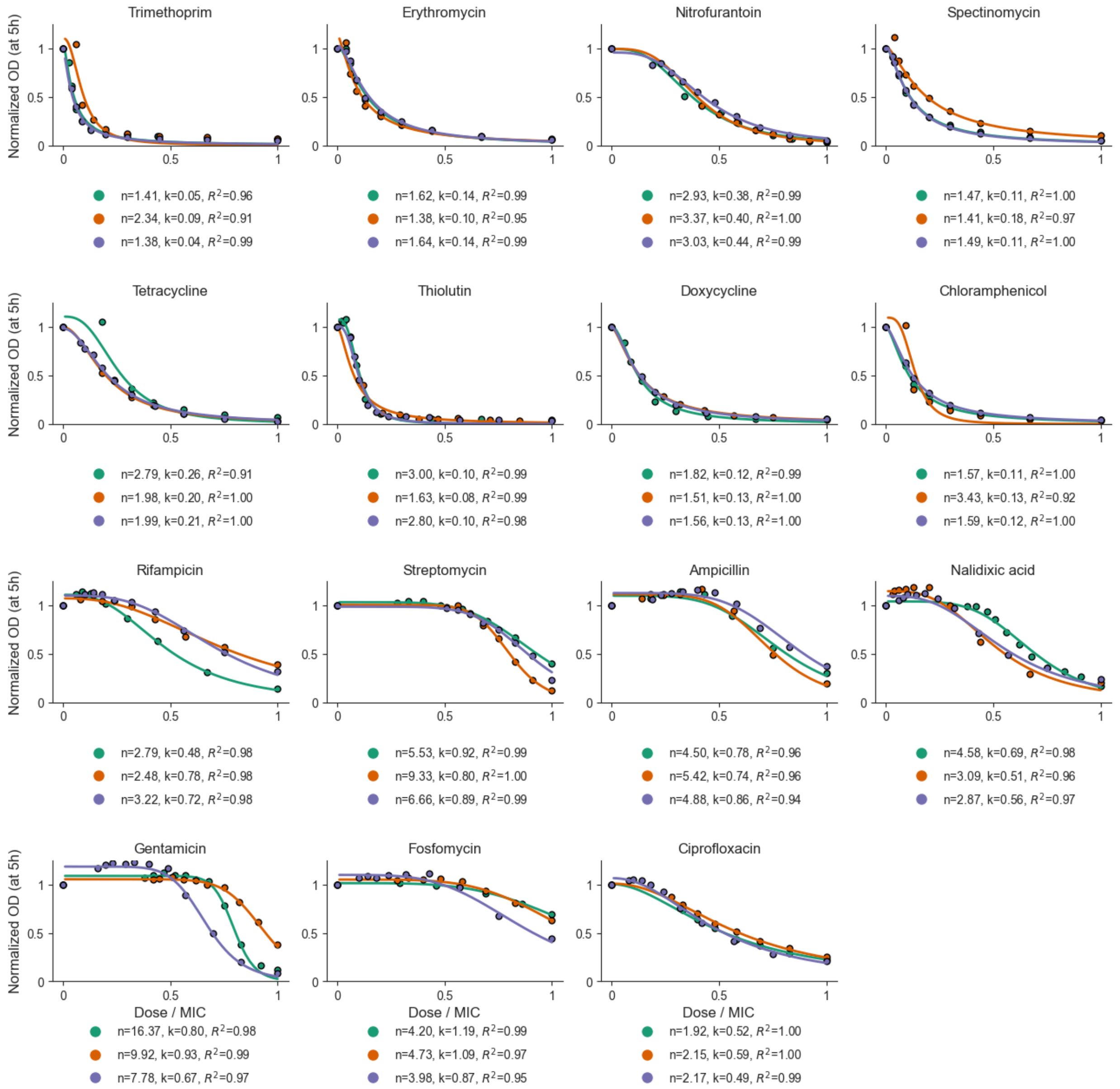

Figure S5. Biological repeats of the yield at 5h of treatment. Colors indicate separate biological replicates. The fitted Hill coefficients k, n, and goodness of fit (R^2) are indicated for each biological replicate.

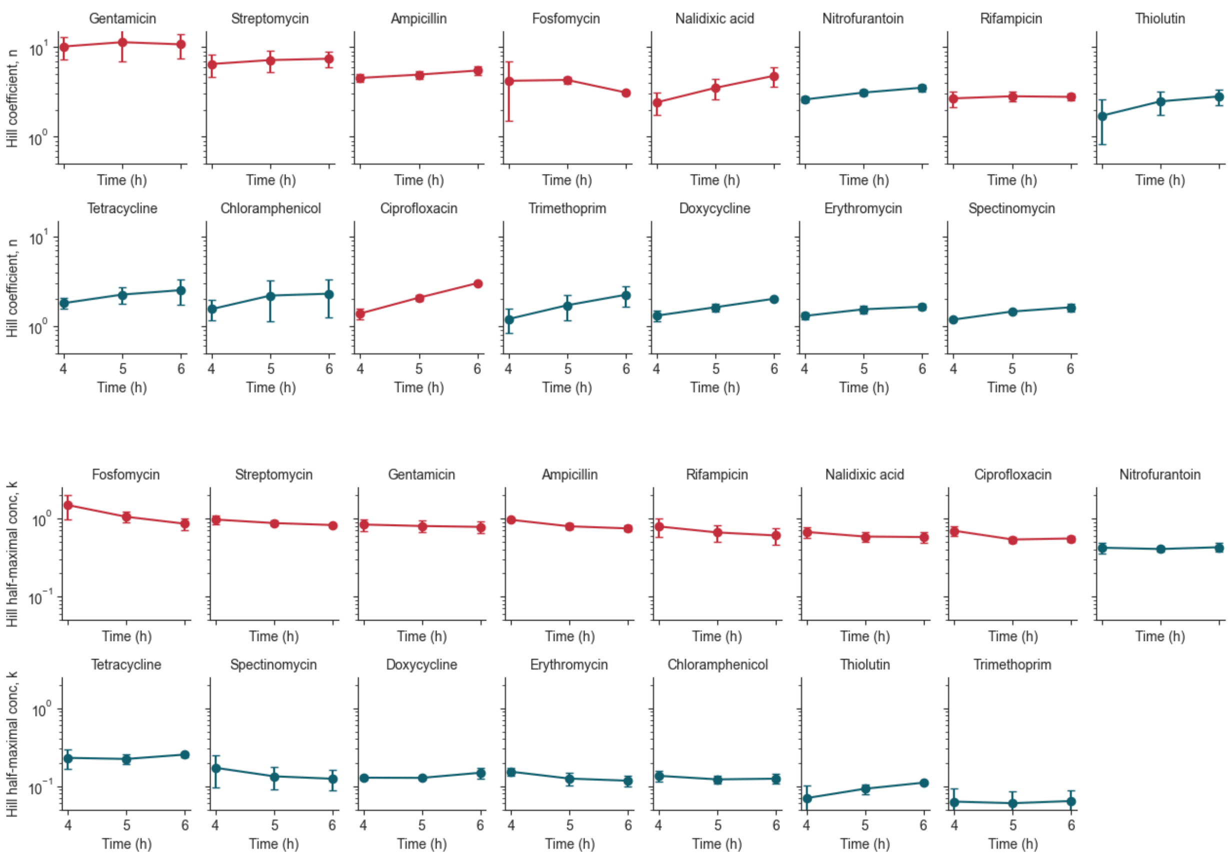

Figure S6. Hill coefficients n and k are insensitive to the time of sampling the growth yield across all antibiotics. Error bars are SD of three biological replicates, each with 4-6 technical replicates. Traces are colored by drug class.

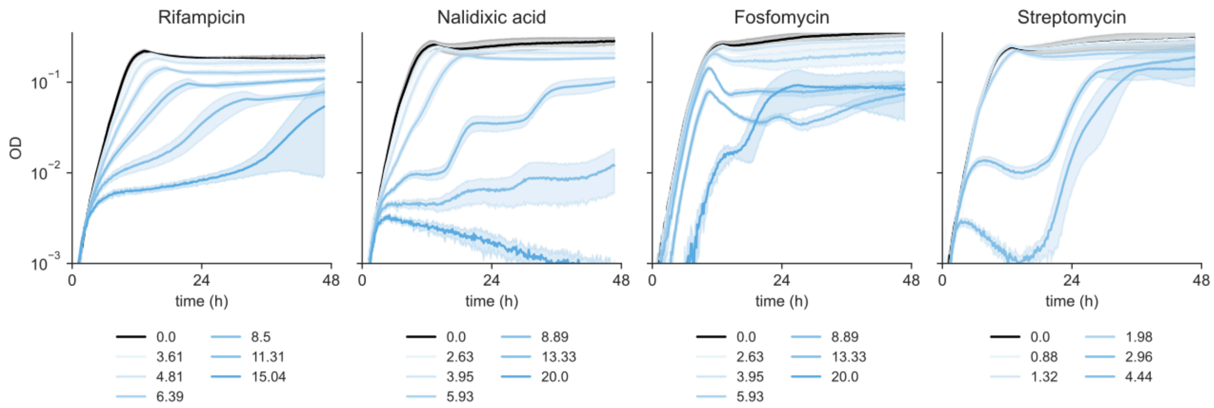

Figure S7. Growth trajectories of the MG1655 strain in M9+glucose for 48 h.
Data are presented as means for 4-6 technical replicates per curve. Concentrations are indicated in µg/ml.

Table S1. Minimal inhibitory concentrations for the antibiotics used in this study.
See Methods for the determination of MIC and the antibiotic class.

**Antibiotic**	**MIC ± SEM**	**Growth pattern according to Fig.1**	**Antibiotic class according to MBC/MIC ratio**
Ampicillin	7.5 ± 1 ug/ml	delayed	cidal
Chloramphenicol	6 ± 0 ug/ml	immediate	static
Ciprofloxacin	15 ± 3.5 ng/ml	delayed	cidal
Doxycycline	1.1 ± 0.4 ug/ml	immediate	static
Erythromycin	89 ± 0 ug/ml	immediate	static
Fosfomycin	18 ± 4 ug/ml	delayed	cidal
Gentamicin	0.6 ± 0.08 ug/ml	delayed	cidal
Nalidixic acid	8 ± 0.6 ug/ml	delayed	cidal
Nitrofurantoin	7.3 ± 0.2 ug/ml	immediate	cidal
Rifampicin	9 ± 1 ug/ml	delayed	static
Spectinomycin	67 ± 0 ug/ml	immediate	static
Streptomycin	5.5 ± 1 ug/ml	delayed	cidal
Tetracycline	2.5 ± 0.5 ug/ml	immediate	static
Thiolutin	2.6 ± 0.4 ug/ml	immediate	static
Trimethoprim	2.3 ± 1 ug/ml	immediate	static

Table S2. Hill coefficients for the antibiotics.
Mean n, k, alpha ± SEM reported for 12-18 technical repeats across 3 biological repeats.

**Drug**	**n**	**k**	**α**
Ampicillin	4.93 ± 0.27	0.79 ± 0.04	1.12 ± 0.01
Chloramphenicol	2.20 ± 0.62	0.12 ± 0.01	1.03 ± 0.03
Ciprofloxacin	2.08 ± 0.08	0.54 ± 0.03	1.02 ± 0.02
Doxycycline	1.63 ± 0.10	0.13 ± 0.00	1.01 ± 0.00
Erythromycin	1.54 ± 0.08	0.12 ± 0.01	1.07 ± 0.04
Fosfomycin	4.30 ± 0.22	1.05 ± 0.09	1.05 ± 0.02
Gentamicin	11.36 ± 2.58	0.80 ± 0.08	1.11 ± 0.03
Nalidixic acid	3.51 ± 0.54	0.59 ± 0.03	1.10 ± 0.03
Nitrofurantoin	3.11 ± 0.13	0.41 ± 0.02	0.98 ± 0.01
Rifampicin	2.83 ± 0.22	0.66 ± 0.09	1.10 ± 0.01
Spectinomycin	1.46 ± 0.03	0.13 ± 0.03	1.03 ± 0.00
Streptomycin	7.17 ± 1.13	0.87 ± 0.04	1.02 ± 0.01
Tetracycline	2.25 ± 0.27	0.22 ± 0.02	1.03 ± 0.01
Thiolutin	2.48 ± 0.43	0.09 ± 0.01	1.03 ± 0.02
Trimethoprim	1.71 ± 0.31	0.06 ± 0.01	1.07 ± 0.01

Table S3. Fitted parameters and goodness of fit for the time-to-deviate model (Fig. 5).
Mean fitted parameters τ, $\frac{y_c}{\alpha}$ ± SD for the fits, pooled data across 3 biological repeats.

**Drug**	$\tau\ [h]$	$\frac{y_c}{\alpha}\ [MIC]$	**RMSE [h]**	**MAE [h]**
Ampicillin	2.7 ± 0.08	0.51 ± 0.01	0.23	0.19
Ciprofloxacin	2.8 ± 0.09	0.06 ± 0.03	0.21	0.18
Fosfomycin	3.7 ± 0.06	0.31 ± 0.002	1.19	0.98
Gentamicin	1.82 ± 0.05	0.55 ± 0.01	0.87	0.60
Nalidixic acid	2.84 ± 0.1	0.24 ± 0.04	0.21	0.17
Rifampicin	2.63 ± 0.06	0.27 ± 0.01	0.56	0.38
Streptomycin	1.32 ± 0.05	0.54 ± 0.01	0.24	0.20
